# Clinical Overview of Leber Hereditary Optic Neuropathy

**DOI:** 10.15388/Amed.2022.29.1.19

**Published:** 2022-06-29

**Authors:** Almina Stramkauskaitė, Ieva Povilaitytė, Brigita Glebauskienė, Rasa Liutkevičienė

**Affiliations:** Department of Ophthalmology, Lithuanian University of Health Sciences, Medical Academy, Kaunas, Lithuania; Department of Ophthalmology, Lithuanian University of Health Sciences, Medical Academy, Kaunas, Lithuania; Department of Ophthalmology, Lithuanian University of Health Sciences, Medical Academy, Kaunas, Lithuania; Department of Ophthalmology, Lithuanian University of Health Sciences, Medical Academy, Kaunas, Lithuania; Neuroscience Institute, Lithuanian University of Health Sciences, Medical Academy, Kaunas, Lithuania

**Keywords:** Leber hereditary optic neuropathy, aetiology, clinical features, diagnosis, treatment

## Abstract

Leber hereditary ptic neuropathy (LHON) is a disease of young adults with bilateral, painless, subacute visual loss. The peak age of onset of LHON is in the second and third decades of life. Men are 4 times more likely to be affected than women. In about 25-50% of cases, both eyes are affected simultaneously. In unilateral cases, the other eye is usually affected 2 to 3 months later. Visual acuity deteriorates to counting fingers or worse with a dense central or centrocecal scotoma. In the subacute phase, the optic disc may appear hyperemic with swelling of the peripapillary retinal nerve fibre layer, peripapillary telangiectasias, and increased vascular tortuosity. Ocular coherence tomography of the macula shows marked thinning of the ganglion cell complex even at this stage. The diagnosis of LHON is made in a subject with a consistent clinical history and/or one of three common pathogenic mitochondrial DNA (mtDNA) variants identified by molecular genetic testing. Idebenone was approved by the European Medicines Agency under exceptional circumstances for the treatment of LHON. Current evidence suggests some benefit to vision in a subset of affected individuals treated with idebenone, particularly when treated within the first year of onset of vision loss. In this article, we discuss aetiology, clinical features, diagnosis, differential dignosis, prognosis and treatment.

## Santrauka.

Paveldima Lėberio optinė neuropatija (PLON) jaunų suaugusiųjų liga, pasireiškianti abipusiu, beskausmiu, poūmiu regos praradimu. PLON dažniausiai prasideda antrame ir trečiame gyvenimo dešimtmečiais. Vyrai šia liga serga keturis kartus dažniau nei moterys. Apie 25–50 % atvejų, abi akys pažeidžiamos tuo pačiu metu. Vienpusio regos nervo pažeidimo atvejais kitoje akyje panašūs simptomai pasireiškia po 2–3 mėnesių. Regos aštrumas mažėja iki pirštų skaičiavimo ar dar blogiau, taip pat nustatoma centrinė ar centrocekalinė skotoma. Poūmėje fazėje matomas hipereminis regos nervo diskas kartu su peripapiliniu tinklainės nervinių skaidulų pluošto (TNSP) paburkimu, peripapilinėmis telangiektazijomis, padidėjęs kraujagyslių vingiuotumas. Optinė koherentinė tomografija (OKT) rodo esant žymų ganglinių ląstelių suplonėjimą geltonojoje dėmėje. PLON diagnozė nustatoma remiantis nuoseklia klinikine istorija ir (ar) molekuliniais genetiniais tyrimais, nustačius vieną iš trijų patogenetinių mitochondrinės DNR (mtDNR) mutacijų. Idebenonas buvo patvirtintas Europos vaistų agentūros PLON gydyti. Įrodyta, kad regėjimas pagerėjo daliai pacientų, kai gydymas idebenonu buvo pradėtas per pirmuosus metus nuo regos aštrumo blogėjimo. Šiame straipsnyje aptariama PLON etiologija, klinikiniai požymiai, diagnozė ir gydymas.

## Introduction

Leber hereditary optic neuropathy (LHON) is the most common primary mitochondrial DNA disease, with the majority of patients having one of three primary mtDNA point mutations, namely m.3460G > A (MTND1), m.11778G > A (MTND4), and m.14484T > C (MTND6) [1]. In addition to the three primary mutations, approximately 30 different point mutations have been associated with LHON, which are divided into two groups: the “top-14”, which include the three primary mutations, and a group of cases that occur only in a family or in single cases [2]. LHON is characterized by bilateral, subacute loss of vision due to preferential loss of retinal ganglion cells (RGCs) in the inner retina, resulting in optic nerve degeneration. The dead RGCs are then unable to send visual signals to the brain, resulting in output that is not processed properly, leading to blindness and extreme visual impairment. RGCs loss occurs in about 50% of males and about 10-15% of females. Recovery of lost vision is possible depending on the mutation [3]. LHON is not age-dependent as it affects people in all age groups, but it is reported that males between the ages of 20 and 30 are most commonly affected [4]. The mutation in the mitochondrial genome (mtDNA) occurs in the subunit encoding complex-I (CI) of the electron transport chain, NADH:ubiquinone oxidoreductase, and the mutation usually involves the replacement of a single amino acid. This in turn leads to a lack of energy in neurons, causing neuronal death [5]. Apart from CI dysfunction, impairment of the glutamate transport system and increased oxidative stress also lead to RGCs dysfunction and death [6]. Currently available treatments reduce optic nerve stress to some extent, but do not completely eliminate the condition [7]. Therefore, in this article, we discuss aetiology, clinical features, diagnosis, and treatment.

## Aetiology

LHON is a maternally inherited mitochondrial disorder that results in central scotoma due to thinning of the retinal nerve fiber layer and death of RGCs [8–10]. Because this inherited disease usually involves a mutation in the mtDNA that is passed through maternal inheritance, there is a history of vision loss in maternal relatives in more than half of families. Surprisingly, there is no family history in up to 40% of cases, but this is mainly because tracing is difficult, as de novo mutations are particularly rare [11–13]. The severity of LHON depends on whether the factors are homoplasmic or heteroplasmic [14]. While milder LHON mutations are homoplasmic, more severe CI mutations are both homoplasmic, leading to optic atrophy, and heteroplasmic, leading to basal ganglia degeneration, dystonia, as well as Leigh syndrome [8].

Many pathogenetic mechanisms of LHON are known, including CI dysfunction with decreased adenosine triphosphate (ATP) synthesis. Oxidative phosphorylation (OXPHOS) is responsible for the majority of ATP synthesis, and a chain of five respiratory complexes located at the inner mitochondrial membrane plays a central role in this process. All three missense mutations (ND1, ND4, ND6) of LHON are subunits of complex I. Therefore, damage to the respiratory chain can lead to a lack of ATP synthesis, resulting in RGCs degeneration or death [8,10,15]. Due to apoptotic RGCs, visual signals do not reach the brain, thus the output is not processed properly, resulting in blindness and severe visual damage. Although the visual loss is painless, inflammation of the optic nerves may cause discomfort in rare cases [3,16]. In some studies, the respiratory chain was impaired in patients with the m. 11778G > A mutation, but not in those with the m. 3460G > A mutation. The outcome of respiratory impairment in LHON is controversial and further studies are needed [10].

Apart from the impairment of CI, caused by energetic failure, an impaired respiratory chain can lead to increased production of ROS, so that increased levels of oxidative stress also accounts for the degeneration of the RGCs [6,17]. Mitochondria are responsible for the majority of ROS production, while ROS can damage any cell [17]. Although the primary LHON mutation is a prerequisite, secondary factors are responsible for the risk of vision loss [6]. Cigarette smoking is associated with either the onset or progression of LHON, and heavy smokers are more susceptible to the disease. Heavy drinkers tend to develop an increased risk of vision loss, although the progression of the disease is not as severe as with smoking [18]. Interestingly, some studies report that vitamin B12 deficiency contributes to the development of LHON due to a lack of relevant metabolic cofactors [19].

## Clinical features

Based on the clinical features of LHON, it is generally divided into an acute and a chronic phase, while some studies also describe a presymptomatic phase [20–23]. The latter stage shows ophthalmoscopic changes, such as edema of the retinal nerve fiber layer, tortuosity of the small vessels, and peripapillary microangiopathy [24]. More comprehensive testing may reveal more subtle manifestations of optic nerve dysfunction. According to the literature, asymptomatic carriers of LHON mutation 1178 may have dyschromatopsia with predominantly red/green color discrimination and decreased spatial contrast sensitivity [25]. Not all individuals who are carriers of the mutation, suffer from visual impairment. However, the majority of carriers present with LHON symptoms between the ages of 15 and 35 years, and the disease predominantly affects males, with an estimated male-to-female ratio of 5:1 [26]. The onset of the disease is characterised by acute, painless loss of central vision, with both eyes affected simultaneously in approximately 25% of cases [26]. Although this disease usually occurs bilaterally, it occurs asynchronously. Initially, only one eye is affected, while the other eye becomes damaged within a year. Considering that vision loss is painless, discomfort may rarely occur due to inflammation of the optic nerves [3,4]. A sudden onset of blurred and cloudy vision are the most common initial symptoms of the acute phase of LHON [22,27,28]. Presymptomatic ophthalmoscopic manifestations may persist throughout life in many patients, but once central vision disappears, some new ophthalmoscopic features may appear, such as edema of superior and inferior fibre arcades and axonal loss within the papillomacular bundle (PMB) [24,29]. These fundus changes are visible either before or later after vision loss [30]. Vision deterioration becomes more severe after four to six weeks, with visual acuity typically being 6/60. Loss of colour discrimination and either central or centrocaecal scotoma is also observed [31,32]. Although this phase usually lasts several weeks, approximately 20% to 30% of patients do not show any ophthalmoscopic abnormalities during the acute phase [33]. Later, the edema of the nerve fibres decreases, and the chronic phase is usually reached more than a year after the onset of the disease [34]. Due to the early failure of the PMB, most of the remaining nerve fibers degenerate, resulting in complete optic atrophy, which is more pronounced on the temporal side [23,27,35]. Although affected individuals with the 14484 mutation or aged < 20 years may recover spontaneously, recovery of vision is usually minimal and the prognosis of visual recovery is poor [10,27,33,36]. In the chronic phase, vision loss is usually not progressive, and the majority of patients are considered legally blind [37,38]. Vision loss stabilizes and reaches its nadir within 6-12 months after the onset of the disease [33,36]. When a patient is diagnosed in the late stages and has no maternal history, a brief differential diagnosis is required. It takes at least one month to obtain the results of molecular testing. For this reason, comprehensive testing, including neuroimaging of the retina, optic nerves, chiasm, and optic tracts, should not be delayed in order to make the correct diagnosis and treatment. However, neither gender nor mutation status is responsible for the timing or severity of vision loss [23].

According to the consensus, LHON has the following stages (Table 1):

**Table 1.** Leber hereditary optic neuropathy stages

Asymptomatic (the carriers of the mutation).	Subacute (from the manifestation of LHON to 6 months).	Dynamic (from months 6 to 12).	Chronic (more than 12 months).
Using OCT, thickening of the temporal retinal nerve fibre layer was confirmed in asymptomatic individuals with an LHON-causing mtDNA pathogenic variant, providing evidence that the PMB is particularly vulnerable in LHON [39].	Patients are usually asymptomatic until they develop visual disturbance in the central visual field in the first eye (acute phase). Similar symptoms appear in the other eye on average two to three months later, so that in the majority of cases both eyes are affected within six months. Unilateral optic nerve damage is very rare in patients with LHON, and in such cases another underlying pathologic process should be actively excluded. The most common feature is an enlarging central or centrocecal scotoma. As the size and density of the visual field defect increases, visual acuity deteriorates to the level of finger counting or worse. At this stage, specific changes of the ocular fundus can be observed even in asymptomatic patients: retinal telangiectasias of the peripapillary vessels, edema of the peripapillary retinal nerve fibre layer, which progress and eventually lead to atrophy of the optic nerve [40].	The fundus changes that occur in the subacute stage slowly regress: the edema of the peripapillary retinal nerve fibre layer recedes [40].	At this stage, optic nerve atrophy progresses at different rates: it can be observed from 6 weeks to more than 1 year after the loss of vision. The central or centrocyclic scotoma enlarges. Most patients remain severely visually impaired and meet the legal requirements for blind registration. OCT shows thinning of the retinal fibre layer, especially in the temporal zones [37].

**Figure 1.**
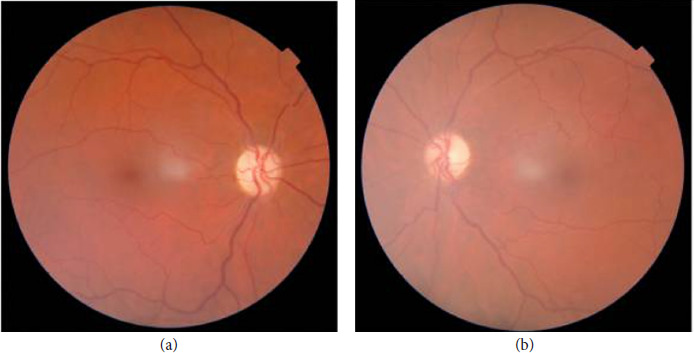
(a, b) Bilateral optic nerve atrophy.

**Figure 2.**
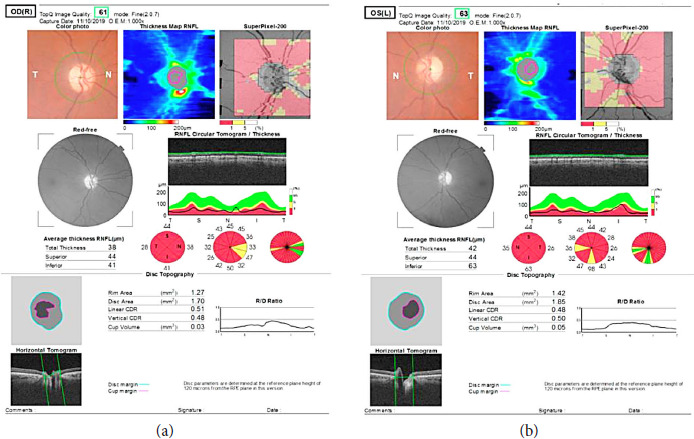
OCT shows thinning of retinal nerve fiber layer.

## Diagnosis

LHON is a poorly diagnosed disease. Correct identification of LHON is critical because of its similarity to several diseases such as isolated optic neuritis or neuromyelitis optica. Attention in the clinical setting is important to correctly identify the disease and exclude other pathologies [41,42]. Molecular genetic testing for pathogenic variants of mtDNA, a multi-gene panel, or complete sequencing of mtDNA are performed to identify LHON. Although the identification of specific mutations in mtDNA plays a central role, other data, such as medical history, fundus, OCT, and magnetic resonance imaging (MRI), are also relevant to the diagnosis of the disease [13]. Some studies recommend that an electrocardiogram be obtained in all patients with LHON because of possible cardiac abnormalities [43–45].

## Differential diagnosis

There is a wide spectrum of acute and chronic optic neuropathies with varying aetiology and variable visual prognosis. The results of ophthalmologic examination, neurologic examination, and imaging studies can aid in diagnosis and treatment. The two main diseases that should be differentiated are: optic neuritis and dominant optic atrophy.

**Optic neuritis (ON).** The main symptoms of ON are very similar to LHON. Symptoms at a young age, mostly in females (ratio of females to males 3:1, in the case of LHON mostly in males), low visual acuity, central scotoma, colour vision loss, pain with eye movements, visual evoked potentials (VEPs) may show a delayed response and reduced P100 amplitude. Rapid recovery and a good therapeutic response to metilprednisolone within a few weeks confirm the diagnosis of optic neuritis. Because of the strong association between optic neuritis and multiple sclerosis, an MRI of the brain should be ordered [46,47].

**Dominant optic atrophy (DOA).** DOA has an insidious onset with mild visual loss beginning in the first two decades of life. Unlike LHON, patients are asymptomatic in the early stages. Patients are often identified only by testing based on a family history or a diagnosis of bilateral optic atrophy. Patients often present with visual acuity of 0.6 or better. Although visual prognosis varies widely at DOA, visual performance is better compared with LHON, with an average visual acuity of 0.3 to 0.1 [23]. Approximately 50-60% of people with DOA have a mutation in OPA -1(3q28-q29) [48,49].

**Ischemic optic neuropathy (ION) and increased intracranial pressure (IIcP).** ION shows optic nerve edema in the acute stage and diffuse optic atrophy in the chronic stage. In patients older than 55 years, arterial ischemic optic neuropathy should be ruled out by determining C-reactive protein and erythrocyte sedimentation rate and consulting a reumatologist who will decide whether to biopsy the temporal artery. If bilateral optic nerve edema is present, the possibility of elevated intracerebral pressure (ICP) should be considered. Symptoms of elevated ICP may include headache, dizziness, nausea, and vomiting. An imaging study should be performed in consultation with a neurologist [46].

**Normal tension glaucoma (NTG)** may have similar clinical features, such as visual acuity loss, paracentral defects, and thinning of the neuroretinal rim on OCT. However, NTG manifests in the older population with an average age of approximately 64 years and older [50]. Arcuate visual field defects, excavation of the inferior rim, and thinning of the inferior optic nerve also distinguish NTG from other mitondrial neuropathies, however genetic testing confirms the LHON diagnosis [51].

**Nutritional and drug-induced optic neuropathies**. Nutrition-induced neuropathies may be caused by deficiencies of vitamins B, B2, B6, B9, B12 [52–54]. Of course, they may also be caused by anorexia nervosa, alcoholism, gastrointestinal problems, or gastrointestinal surgery and must be confirmed by blood tests and by others specialists consultations [55]. Drug-induced optic neuropathies can be caused by ethambutol, linezolid, chloramphenicol, erythromycin and others [23,56–58]. Medical and social history may also be helpful in the differential diagnosis. However, MRI of the brain and orbit must be performed in all cases of optic neuropathy. **Compressive and demyelinating lesions** may cause edematous or atrophic optic nerves. Symptoms of a compressive lesion may include headache and changes in mood, personality, or memory. An MRI of the brain and orbits will confirm the diagnosis [46].

## Treatment

Idebenone (Raxone(®)) is currently the only disease-specific drug approved by the European Medicines Agency in 2015 for the treatment of visual impairment in adolescents and adults with LHON [59,60]. It is a short-chain benzoquinone that is rapidly absorbed and well tolerated [61,62]. Idebenone 300 mg 3 times daily rarely causes adverse symptoms, but the most common side effects include nasopharyngitis, headache, cough, and dizziness [63]. Due to partial structural similarity, idebenone is referred to as a synthetic analogue of coenzyme Q10, but it has different properties and mechanisms of action. Since idebenone has a shorter and less lipophilic tail than coenzyme Q10, its bioactivation occurs mainly in the cytoplasm and does not rely on mitochondrial function. Moreover, idebenone induces respiratory pathways that are independent of mitochondrial CI [61,62,64,65]. Due to its advantageous structure, idebenone can cross membranes and act as a mitochondrial electron transfer agent [61,62,65]. Importantly, idebenone bypasses the disrupted CI and transfers electrons directly to the mitochondrial complex III, resulting in improved energy supply and restoration of inactive but viable retinal ganglion cells, preventing further vision loss and initiating vision restoration [60,65,66]. Many studies have provided convincing evidence that treatment with oral idebenone 900 mg/d for 6 months not only has a beneficial effect on vision, but also improves Tritan colour vision [67,68].

Several studies have been conducted on the treatment of LHON to find the best treatment option for LHON. These include various neuroprotective agents, stem cell therapy, infrared radiation therapy, and even gene therapy [69–71]. The most promising of these methods are gene therapy and stem cell therapy, but more research is needed to evaluate their efficacy. Gene therapy is suitable for LHON because of the easily accessible RGCs layer in the retina. However, inducing genes into the mitochondrial genome is not easy to perform because mitochondria have a double membrane. However, the desired gene can be transferred to the nuclear genome instead. These studies show that the treatment of LHON is promising. Nevertheless, further studies are needed to evaluate adverse effects and determine the longevity of beneficial effects. The use of stem cells to treat LHON is under investigation, but appears promising as well. Mesenchymal stem cells protect RGCs by secreting both neurotrophic factors and anti-inflammatory cytokines. These stem cells were injected into a rat model of glaucoma and a beneficial effect on RGCs axon survival was observed [72].

Bromidine, an α2-agonist used to treat patients with glaucoma, was found to have a stabilizing effect on RGCs. Because the antiapoptotic properties of bromidine act through CI, it would be a perfect treatment for LHON because the major mitochondrial mutations are also found in this complex. However, bromidine is not suitable for prophylaxis of visual disturbances in the second eye, when acute visual disturbance due to LHON is observed in one eye, studies show [73,74].

Some environmental factors such as smoking and alcohol consumption accelerate the risk of vision loss. Therefore, patients should be advised to avoid smoking and minimize alcohol consumption [18,37]. Because vision loss does not usually progress over time, appropriate occupational therapy and low vision aids can improve the quality of life of LHON patients. [37].

## Visual prognosis

Visual prognosis is mutation dependent. The highest chances of visual improvement are observed in patients with mutation 14484T > C, while the lowest chances are observed with mutation 11778G > A [10]. Nevertheless, an early age of onset, a subacute course with slow visual deterioration, and a larger optic disc are associated with a better visual prognosis. [75,76]. However, most patients with LHON experience permanent loss of vision, which negatively affects their quality of life [37].

## Conclusion

LHON is a rare disease characterized by degeneration of RGCs.

LHON is leading to bilateral visual loss with poor prognosis.

The disease is caused by mitochondrial mutations.

Idebenone, gene and stem cells therapy have provided encouraging results.

At the moment, Idebenone is only an approved treatment for LHON.

## References

[1] Sundaramurthy S, SelvaKumar A, Ching J, Dharani V, Sarangapani S, Yu-Wai-Man P. Leber hereditary optic neuropathy-new insights and old challenges. Graefes Arch Clin Exp Ophthalmol. 2021 Sep 1;259(9):2461–72. 33185731 10.1007/s00417-020-04993-1

[2] Cruz-Bermúdez A, Vicente-Blanco RJ, Hernández-Sierra R, Montero M, Alvarez J, Manrique MG, et al. Functional Characterization of Three Concomitant MtDNA LHON Mutations Shows No Synergistic Effect on Mitochondrial Activity. PLoS One. 2016 Jan 1;11(1). 10.1371/journal.pone.0146816 PMC4718627 26784702

[3] Levin LA. Mechanisms of retinal ganglion specific-cell death in leber hereditary optic neuropathy. Trans Am Ophthalmol Soc. 2007;105:379–91. 18427623 PMC2258117

[4] Vergani L, Martinuzzi A, Carelli V, Cortelli P, Montagna P, Schievano G, et al. MtDNA mutations associated with Leber’s hereditary optic neuropathy: studies on cytoplasmic hybrid (cybrid) cells. Biochem Biophys Res Commun. 1995 May 25;210(3):880–8. 7763260 10.1006/bbrc.1995.1740

[5] Kirches E. LHON: Mitochondrial Mutations and More. Curr Genomics. 2011 Feb 7;12(1):44–54. 21886454 10.2174/138920211794520150 PMC3129042

[6] Zhuo Y, Luo H, Zhang K. Leber hereditary optic neuropathy and oxidative stress. Vol. 109, Proceedings of the National Academy of Sciences of the United States of America. National Academy of Sciences; 2012. p. 19882–3. 10.1073/pnas.1218953109 PMC3523847 23197830

[7] Manickam A, Michael M, Ramasamy S. Mitochondrial genetics and therapeutic overview of Leber’s hereditary optic neuropathy. Vol. 65, Indian Journal of Ophthalmology. Medknow Publications; 2017. p. 1087–92. 10.4103/ijo.IJO_358_17 PMC5700573 29133631

[8] Wallace DC, Lott MT. Leber Hereditary Optic Neuropathy: Exemplar of an mtDNA Disease. Handb Exp Pharmacol. 2017;240. 10.1007/164_2017_2 28233183

[9] Qu J, Li R, Zhou X, Tong Y, Lu F, Qian Y, et al. The Novel A4435G Mutation in the Mitochondrial tRNAMet May Modulate the Phenotypic Expression of the LHON-Associated ND4 G11778A Mutation. Invest Ophthalmol Vis Sci. 2006 Feb 1;47(2):475–83. 16431939 10.1167/iovs.05-0665

[10] Yu-Wai-Man P, Griffiths PG, Hudson G, Chinnery PF. Inherited mitochondrial optic neuropathies. J Med Genet. 2009 Mar;46(3):145–58. 19001017 10.1136/jmg.2007.054270 PMC2643051

[11] Man PYW, Griffiths PG, Brown DT, Howell N, Turnbull DM, Chinnery PF. The epidemiology of Leber hereditary optic neuropathy in the North East of England. Am J Hum Genet. 2003 Feb 1;72(2):333–9. 12518276 10.1086/346066 PMC379226

[12] Biousse V, Brown, Newman NJ, Allen JC, Rosenfeld J, Meola G, et al. De novo 14484 mitochondrial DNA mutation in monozygotic twins discordant for Leber’s hereditary optic neuropathy. Neurology. 1997;49(4):1136–38. 9339703 10.1212/wnl.49.4.1136

[13] Yu-Wai-Man P, Chinnery PF. Leber Hereditary Optic Neuropathy. GeneReviews®. 2021 Mar 11

[14] Zeviani M, Di Donato S. Mitochondrial disorders. Brain. 2004 Oct;127(Pt 10):2153–72. 15358637 10.1093/brain/awh259

[15] Ji Y, Zhang J, Yu J, Wang Y, Lu Y, Liang M, et al. Contribution of mitochondrial ND1 3394T>C mutation to the phenotypic manifestation of Leber’s hereditary optic neuropathy. Hum Mol Genet. 2019;28(9):1515–29. 30597069 10.1093/hmg/ddy450

[16] Newman NJ, Lott MT, Wallace DC. The clinical characteristics of pedigrees of Leber’s hereditary optic neuropathy with the 11778 mutation. Am J Ophthalmol. 1991;111(6):750–62. 2039048 10.1016/s0002-9394(14)76784-4

[17] Kannan K, Jain SK. Oxidative stress and apoptosis. Pathophysiol Off J Int Soc Pathophysiol. 2000 Sep;7(3):153–63. 10.1016/s0928-4680(00)00053-5 10996508

[18] Kirkman MA, Yu-Wai-Man P, Korsten A, Leonhardt M, Dimitriadis K, De Coo IF, et al. Gene-environment interactions in Leber hereditary optic neuropathy. Brain. 2009;132(Pt 9):2317–26. 19525327 10.1093/brain/awp158 PMC2732267

[19] Pott JWR, Wong KH. Leber’s hereditary optic neuropathy and vitamin B12 deficiency. Graefe’s Arch Clin Exp Ophthalmol 2006 24410. 2006 Mar 8;244(10):1357–9. 10.1007/s00417-006-0269-7 16523300

[20] Bi R, Logan I, Yao YG. Leber Hereditary Optic Neuropathy: A Mitochondrial Disease Unique in Many Ways. Handb Exp Pharmacol. 2017;240. 10.1007/164_2016_1 27787713

[21] Barboni P, Savini G, Valentino ML, Montagna P, Cortelli P, De Negri AM, et al. Retinal nerve fiber layer evaluation by optical coherence tomography in Leber’s hereditary optic neuropathy. Ophthalmology. 2005 Jan;112(1):120–6. 15629831 10.1016/j.ophtha.2004.06.034

[22] Nikoskelainen E, Nummelin K, Hoyt WF, Schatz H. Fundus findings in Leber’s hereditary optic neuroretinopathy. III. Fluorescein angiographic studies. Arch Ophthalmol (Chicago, Ill 1960). 1984;102(7):981–9. 6743093 10.1001/archopht.1984.01040030783017

[23] Yu-Wai-Man P, Griffiths PG, Chinnery PF. Mitochondrial optic neuropathies - Disease mechanisms and therapeutic strategies. Vol. 30, Progress in Retinal and Eye Research. Elsevier; 2011. p. 81–114. 10.1016/j.preteyeres.2010.11.002 PMC3081075 21112411

[24] Nikoskelainen E, Hoyt WF. Ophthalmoscopic findings in Leber’s hereditary optic neuropathy. II. The fundus findings in the affected family members. Arch Ophthalmol (Chicago, Ill 1960). 1983;101(7):1059–68. 10.1001/archopht.1983.01040020061011 6870629

[25] Quiros PA, Torres RJ, Salomao S, Berezovsky A, Carelli V, Sherman J, et al. Colour vision defects in asymptomatic carriers of the Leber’s hereditary optic neuropathy (LHON) mtDNA 11778 mutation from a large Brazilian LHON pedigree: A case-control study. Br J Ophthalmol. 2006 Feb;90(2):150–3. 16424523 10.1136/bjo.2005.074526 PMC1860163

[26] Seedorff T. The inheritance of Leber’s disease. A genealogical follow-up study. Acta Ophthalmol. 1985;63(2):135–45. 4003041 10.1111/j.1755-3768.1985.tb01526.x

[27] Newman NJ. Hereditary optic neuropathies: from the mitochondria to the optic nerve. Am J Ophthalmol. 2005;140(3):517.e1-517.e9. 16083845 10.1016/j.ajo.2005.03.017

[28] Khanh Vu TH, Zhu R, Yang L, Chen DF. Optic Nerve Structure and Pathologies. Pathobiol Hum Dis A Dyn Encycl Dis Mech. 2014 Jan 1;2115–25.

[29] Smith JL, Hoyt WF, Susac JO. Ocular fundus in acute Leber optic neuropathy. Arch Ophthalmol (Chicago, Ill 1960). 1973;90(5):349–54. /10.1001/archopht.1973.01000050351002 4746084

[30] Chalmers RM, Harding AE. A case-control study of Leber’s hereditary optic neuropathy. Brain. 1996 Oct;119 (Pt 5)(5):1481–6. 8931573 10.1093/brain/119.5.1481

[31] Wakakura M, Yokoe J. Evidence for preserved direct pupillary light response in Leber’s hereditary optic neuropathy. Br J Ophthalmol. 1995;79(5):442–6. 7612556 10.1136/bjo.79.5.442 PMC505132

[32] Kawasaki A, Herbst K, Sander B, Milea D. Selective wavelength pupillometry in Leber hereditary optic neuropathy. Clin Experiment Ophthalmol. 2010 Apr;38(3):322–4. 20447133 10.1111/j.1442-9071.2010.02212.x

[33] Riordan-eva P, Sanders MD, Govan GG, Sweeney MG, Costa JD, Harding AE. The clinical features of Leber’s hereditary optic neuropathy defined by the presence of a pathogenic mitochondrial DNA mutation. Brain. 1995 Apr;118 ( Pt 2(2):319–37. 7735876 10.1093/brain/118.2.319

[34] Yu-Wai-Man P, Newman NJ, Carelli V, La Morgia C, Biousse V, Bandello FM, et al. Natural history of patients with Leber hereditary optic neuropathy—results from the REALITY study. Eye 2021. 2021 Apr 28;20:1–9. 10.1038/s41433-021-01535-9 PMC8956580 33911213

[35] Mashima Y, Kimura I, Yamamoto Y, Ohde H, Ohtake Y, Tanino T, et al. Optic disc excavation in the atrophic stage of Leber’s hereditary optic neuropathy: comparison with normal tension glaucoma. Graefes Arch Clin Exp Ophthalmol. 2003 Feb 1;241(2):75–80. 12605258 10.1007/s00417-002-0598-0

[36] Nikoskelainen EK, Huoponen K, Juvonen V, Lamminen T, Nummelin K, Savontaus ML. Ophthalmologic findings in Leber hereditary optic neuropathy, with special reference to mtDNA mutations. Ophthalmology. 1996;103(3):504–14. /8600429 10.1016/s0161-6420(96)30665-9

[37] Kirkman MA, Korsten A, Leonhardt M, Dimitriadis K, de Coo IF, Klopstock T, et al. Quality of life in patients with leber hereditary optic neuropathy. Invest Ophthalmol Vis Sci. 2009;50(7):3112–5. 19255150 10.1167/iovs.08-3166

[38] Yu-Wai-Man P, Votruba M, Moore AT, Chinnery PF. Treatment strategies for inherited optic neuropathies: past, present and future. Eye (Lond). 2014;28(5):521–37. 24603424 10.1038/eye.2014.37 PMC4017118

[39] Savini G, Barboni P, Valentino ML, Montagna P, Cortelli P, De Negri AM, et al. Retinal nerve fiber layer evaluation by optical coherence tomography in unaffected carriers with Leber’s hereditary optic neuropathy mutations. Ophthalmology. 2005 Jan;112(1):127–31. 15629832 10.1016/j.ophtha.2004.09.033

[40] Nikoskelainen E. K. Clinical Picture of LHON. Clin Neurosci. 1994;2:115–20.

[41] McClelland CM, Van Stavern GP, Tselis AC. Leber hereditary optic neuropathy mimicking neuromyelitis optica. J Neuroophthalmol. 2011 Sep;31(3):265–8. 21734595 10.1097/WNO.0b013e318225247b

[42] Filatov A, Khanni JL, Espinosa PS. Leber Hereditary Optic Neuropathy: Case Report and Literature Review. Cureus. 2020 Apr 20;12(4). 10.7759/cureus.7745 PMC7241220 32454526

[43] Acaroǧlu G, Kansu T, Doǧulu ÇF. Visual recovery patterns in children with Leber’s hereditary optic neuropathy. Int Ophthalmol. 2001;24(6):349–55. 14750573 10.1023/b:inte.0000006855.48323.f1

[44] Orssaud C. Cardiac Disorders in Patients With Leber Hereditary Optic Neuropathy. J Neuroophthalmol. 2018;38(4):466–9. 29384800 10.1097/WNO.0000000000000623

[45] Newman NJ. Treatment of hereditary optic neuropathies. Nat Rev Neurol. 2012;8(10):545–56. 22945544 10.1038/nrneurol.2012.167

[46] Pilz YL, Bass SJ, Sherman J. A Review of Mitochondrial Optic Neuropathies: From Inherited to Acquired Forms. J Optom. 2017 Oct 1;10(4):205–14. 28040497 10.1016/j.optom.2016.09.003 PMC5595256

[47] Kupersmith MJ, Weiss PA, Carr RE. The Visual-Evoked Potential in Tobacco-Alcohol and Nutritional Amblyopia. Am J Ophthalmol. 1983 Mar 1;95(3):307–14. 6829677 10.1016/s0002-9394(14)78298-4

[48] Alexander C., Votruba M. PUE. Identification of the gene responsible for dominant optic atrophy (OPA1) on chromosome 3q28. xt. 2000;67:40.

[49] Delettre C, Lenaers G, Griffoin JM, Gigarel N, Lorenzo C, Belenguer P, et al. Nuclear gene OPA1, encoding a mitochondrial dynamin-related protein, is mutated in dominant optic atrophy. Nat Genet. 2000 Oct;26(2):207–10. 11017079 10.1038/79936

[50] Alward WLM, Feldman F, Trope G, Cashwell LF, Wilensky J, Geijssen HC, et al. Natural history of normal-tension glaucoma. Ophthalmology. 2001;108(2):247–53. 11158794 10.1016/s0161-6420(00)00518-2

[51] Buono LM, Foroozan R, Sergott RC, Savino PJ. Is normal tension glaucoma actually an unrecognized hereditary optic neuropathy? New evidence from genetic analysis. Curr Opin Ophthalmol. 2002 Dec;13(6):362–70. 12441838 10.1097/00055735-200212000-00004

[52] Sadun AA. Metabolic optic neuropathies. Semin Ophthalmol. 2002 Mar;17(1):29–32. 15513453 10.1076/soph.17.1.29.10290

[53] Hsu CT, Miller NR, Wray ML. Optic neuropathy from folic acid deficiency without alcohol abuse. Ophthalmologica. 2002;216(1):65–7. 11901292 10.1159/000048300

[54] Orssaud C, Roche O, Dufier JL. Nutritional optic neuropathies. J Neurol Sci. 2007 Nov 15;262(1–2):158–64. 17707410 10.1016/j.jns.2007.06.038

[55] Thompson R.E. FJL. Nutritional amblyopia associated with jejunoileal bypass surgery. Ann Ophthalmol. 1982;14:848–50. 7181347

[56] Fraunfelder FW, Sadun AA, Wood T. Update on ethambutol optic neuropathy. Expert Opin Drug Saf. 2006 Sep;5(5):615–8. 16907651 10.1517/14740338.5.5.615

[57] Joshi L, Taylor SRJ, Large O, Yacoub S, Lightman S. A case of optic neuropathy after short-term linezolid use in a patient with acute lymphocytic leukemia. Clin Infect Dis. 2009 Apr 1;48(7). 10.1086/597298 19231981

[58] Godel V, Nemet P, Lazar M. Chloramphenicol optic neuropathy. Arch Ophthalmol (Chicago, Ill 1960);1980;98(8):1417–21. 10.1001/archopht.1980.01020040269011 7417077

[59] CHMP. ANNEX I SUMMARY OF PRODUCT CHARACTERISTICS.

[60] CHMP. Committee for Medicinal Products for Human Use (CHMP) Assessment report. 2015;

[61] Suno M, Nagaoka A. Inhibition of lipid peroxidation by idebenone in brain mitochondria in the presence of succinate. Arch Gerontol Geriatr. 1989;8(3):291–7. /2764644 10.1016/0167-4943(89)90010-1

[62] Haefeli RH, Erb M, Gemperli AC, Robay D, Fruh I, Anklin C, et al. NQO1-Dependent Redox Cycling of Idebenone: Effects on Cellular Redox Potential and Energy Levels. PLoS One. 2011 Mar 31;6(3):e17963. 21483849 10.1371/journal.pone.0017963 PMC3069029

[63] Klopstock T, Yu-Wai-Man P, Dimitriadis K, Rouleau J, Heck S, Bailie M, et al. A randomized placebo-controlled trial of idebenone in Leber’s hereditary optic neuropathy. Brain. 2011;134(Pt 9):2677–86. 21788663 10.1093/brain/awr170 PMC3170530

[64] Gueven N, Woolley K, Smith J. Border between natural product and drug: Comparison of the related benzoquinones idebenone and coenzyme Q10. Redox Biol. 2015 Apr 1;4:289–95. 25625583 10.1016/j.redox.2015.01.009 PMC4803797

[65] Heitz FD, Erb M, Anklin C, Robay D, Pernet V, Gueven N. Idebenone protects against retinal damage and loss of vision in a mouse model of Leber’s hereditary optic neuropathy. PLoS One. 2012 Sep 18;7(9). 10.1371/journal.pone.0045182 PMC3445472 23028832

[66] Lyseng-Williamson KA. Idebenone: A Review in Leber’s Hereditary Optic Neuropathy. Drugs. 2016 May 1;76(7):805–13. 27071925 10.1007/s40265-016-0574-3

[67] Catarino CB, Klopstock T. Use of Idebenone for the Treatment of Leber’s Hereditary Optic Neuropathy. J Inborn Errors Metab Screen. 2017 Jan 18;5:232640981773111.

[68] Rudolph G, Dimitriadis K, Büchner B, Heck S, Al-Tamami J, Seidensticker F, et al. Effects of idebenone on color vision in patients with leber hereditary optic neuropathy. J Neuroophthalmol. 2013 Mar;33(1):30–6. 23263355 10.1097/WNO.0b013e318272c643 PMC3658961

[69] Carelli V, Carbonelli M, De Coo IF, Kawasaki A, Klopstock T, Lagrèze WA, et al. International Consensus Statement on the Clinical and Therapeutic Management of Leber Hereditary Optic Neuropathy. J Neuroophthalmol. 2017;37(4):371–81. 28991104 10.1097/WNO.0000000000000570

[70] Weiss JN, Levy S, Benes SC. Stem Cell Ophthalmology Treatment Study (SCOTS): bone marrow-derived stem cells in the treatment of Leber’s hereditary optic neuropathy. Neural Regen Res. 2016 Oct 1;11(10):1685–94. 27904503 10.4103/1673-5374.193251 PMC5116851

[71] Yuan J, Zhang Y, Liu H, Wang D, Du Y, Tian Z, et al. Seven-Year Follow-up of Gene Therapy for Leber’s Hereditary Optic Neuropathy. Ophthalmology. 2020 Aug 1;127(8):1125–7. 32284191 10.1016/j.ophtha.2020.02.023

[72] Dahlmann-Noor A, Vijay S, Jayaram H, Limb A, Khaw PT. Current approaches and future prospects for stem cell rescue and regeneration of the retina and optic nerve. Can J Ophthalmol. 2010;45(4):333–41. 20648090 10.3129/i10-077

[73] Saylor M, McLoon LK, Harrison AR, Lee MS. Experimental and clinical evidence for brimonidine as an optic nerve and retinal neuroprotective agent: an evidence-based review. Arch Ophthalmol (Chicago, Ill 1960). 2009 Apr;127(4):402–6. 10.1001/archophthalmol.2009.9 19365015

[74] Newman NJ, Biousse V, David R, Bhatti MT, Hamilton SR, Farris BK, et al. Prophylaxis for second eye involvement in leber hereditary optic neuropathy: an open-labeled, nonrandomized multicenter trial of topical brimonidine purite. Am J Ophthalmol. 2005;140(3):407.e1-407.e11. 16083844 10.1016/j.ajo.2005.03.058

[75] Barboni P, Savini G, Valentino ML, La Morgia C, Bellusci C, De Negri AM, et al. Leber’s hereditary optic neuropathy with childhood onset. Invest Ophthalmol Vis Sci. 2006 Dec;47(12):5303–9. 17122117 10.1167/iovs.06-0520

[76] Ramos C do VF, Bellusci C, Savini G, Carbonelli M, Berezovsky A, Tamaki C, et al. Association of Optic Disc Size with Development and Prognosis of Leber’s Hereditary Optic Neuropathy. Invest Ophthalmol Vis Sci. 2009 Apr 1;50(4):1666–74. 19098324 10.1167/iovs.08-2695

